# Associations between neighbourhood deprivation, ethnicity and maternal health outcomes in England: a nationwide cohort study using routinely collected healthcare data

**DOI:** 10.1136/jech-2024-222060

**Published:** 2024-06-04

**Authors:** Dorothea Geddes-Barton, Rema Ramakrishnan, Marian Knight, Raph Goldacre

**Affiliations:** Nuffield Department of Population Health, University of Oxford, Oxford, UK

**Keywords:** PUBLIC HEALTH, OBSTETRICS, SOCIAL CLASS, MATERNAL HEALTH

## Abstract

**Background:**

In the United Kingdom, pregnant women who live in the most deprived areas have two times the risk of dying than those who live in the least deprived areas. There are even greater disparities between women from different ethnic groups. The aim of this study was to investigate the role of area-based deprivation and ethnicity in the increased risk of severe maternal morbidity (SMM), in primiparous women in England.

**Methods:**

A retrospective nationwide population study was conducted using English National Hospital Episode Statistics Admitted Patient Care database. All primiparous women were included if they gave birth in an National Healthcare Service (NHS) hospital in England between 1 January 2016 and 31 December 2021. Logistic regression was used to examine the relative odds of SMM by Index of Multiple Deprivation and ethnicity, adjusting for age and health behaviours, medical and psychological factors.

**Results:**

The study population comprised 1 178 756 primiparous women. Neighbourhood deprivation increased the risk of SMM at the time of childbirth. In the fully adjusted model, there was a linear trend (p=0.001) between deprivation quintile and the odds of SMM. Being from a minoritised ethnic group also independently increased the risk of SMM, with black or black British African women having the highest risk, adjusted OR 1.84 (95% CI 1.70 to 2.00) compared with white women. There was no interaction between deprivation and ethnicity (p=0.49).

**Conclusion:**

This study has highlighted that neighbourhood deprivation and ethnicity are important, independently associated risk factors for SMM.

**WHAT IS ALREADY KNOWN ON THIS TOPIC**

- Neighbourhood deprivation and being from a minoritised ethnicity are associated with an increased risk of maternal mortality in the United Kingdom (UK). However, there is a lack of quantitative evidence illustrating the relationship between ethnicity and neighbourhood deprivation and the role of this relationship on the risk of severe maternal morbidity (SMM).
- A scoping literature review was performed aiming to examine any existing literature, which has documented the relationship between deprivation/socioeconomic status and maternal morbidity limited to the UK. Medline was searched on 1 July 2022. An example search term included “Socioeconomic Factors (Mesh)” OR “Deprivation” OR “Socioeconomic” OR “Disparities” OR “Poverty” OR “Standards of living” OR “Social inequality” AND “Maternal mortality (MESH)” OR “Maternal near-miss” OR “SMM” AND “England” OR “United Kingdom” OR “UK” OR “Britain” OR “London” OR “Birmingham”.
- There are currently no studies which examine the relationship between neighbourhood deprivation and a composite outcome of SMM in the UK, although the studies which have looked at individual morbidity events have shown a significant relationship. Being from a minoritised ethnicity has been shown to be a risk factor for SMM, but there is a lack of quantitative research exploring the independent and potentially interactive associations of both ethnicity and deprivation on the risk of SMM.

**WHAT THIS STUDY ADDS**

- This study that includes every primiparous woman who gave birth in an National Healthcare Service (NHS) hospital in England in 2016–2021, has shown that women have increased odds of SMM if they live in the most deprived areas of the country or if they are from a minoritised ethnic group. Both socioeconomic deprivation and ethnicity are independently associated with SMM rather than one being mostly a surrogate marker for the other, and no ethnic group appeared to be more affected by the higher rates of SMM associated with deprivation.

**HOW THIS STUDY MIGHT AFFECT RESEARCH, PRACTICE OR POLICY**

- This study, alongside the existing evidence, highlights to clinicians, policymakers and women, that urgent action is needed to reduce socioeconomic and ethnic inequalities in maternal health outcomes, and that these risk factors need to be addressed independently. Alongside the development of targeted and evidenced-based interventions, there is an urgent need to address the underlying systems and structures which are driving the increased risk for disadvantaged groups.

## Introduction

The social determinants of health (SDOH) defined as the conditions in which people are born, live, and work, are significant drivers of disease risk and susceptibility. These determinants include income, employment, education, access to safe and affordable housing, and access to nutritious foods. The differences in social position and power shape identities and access in society.^1^

In the UK, such determinants have been shown to be important risk factors for maternal mortality, with women who live in the most deprived areas being more than twice as likely to die than those who live in the least.^2^ However, for every woman who dies, many more experience a life-threatening event, or ‘severe maternal morbidity (SMM)’ during pregnancy, childbirth or postpartum. Studies^3–5^ conducted in other high-income countries have shown a convincing relationship between neighbourhood deprivation and SMM. There is currently a paucity of research for the role of neighbourhood deprivation and risk of SMM in the UK.

Minoritised ethnicities are disproportionally represented in the most deprived areas in the UK. Furthermore, in the UK, being from a minoritised ethnic group has been shown to be a risk factor for both maternal mortality and morbidity.^2 6 7^ However, it is currently unclear whether the differences in outcome between white women and those from minoritised ethnic groups persist after adjusting for socioeconomic deprivation. In addition, the most recent confidential enquiry into perinatal deaths in the UK^8^ showed that some ethnic groups are more affected by the higher rates of stillbirth associated with deprivation. No study has explored whether this is also the case for SMM.

This study, therefore, aimed to describe whether either living in the most deprived areas or being from a minoritised ethnic group is associated with SMM at the time of giving birth in England, whether one risk factor is mainly a surrogate marker for the other, and whether the association between living in deprivation and SMM is greater in certain ethnic groups. Additionally, this study aimed to examine the role of individual confounding factors to understand what may be accounting for any differences in more detail.

## Methods

### Study design

A retrospective nationwide population study was conducted, using the English National Hospital Episode Statistics Admitted Patient Care (HES APC) database.

### Data source

The HES APC is a national administrative hospital database that includes records of all hospital admissions including pregnancy and birth-specific information in an additional maternity section.^9^ For this study, data were extracted on all childbirth episodes in England between 1 January 2016 and 31 December 2021 and linked hospital admissions for any cause from the 1 Januaray 2003. Further details of the HES APC database have been described elsewhere.^10^ Diagnostic information is coded using the International Classification of Disease 10th addition and operative procedures are coded using UK Office for Population Censuses and Surveys classification, fourth revision.^10^

### Ethics committee approval, data availability and reporting

This study is reported according to recommendations in the Strengthening the Reporting of Observational Studies in Epidemiology Guidelines.^11^

### PECOS (Population, Exposure, Comparison, Outcome, Study design)

This study included all primiparous women aged 10–54 who gave birth between 1 January 2016 and 31 December 2021, in hospital in England, with a gestational age at childbirth of greater than 20 weeks. The first exposure was defined based on the 2019 release of the Index of Multiple Deprivation (IMD).^12^ The IMD is a composite area-based deprivation measure comprising of seven domains based on neighbourhoods of around 1500 residents. A weighted sum of the ranks for each domain is used to calculate an overall IMD score for each neighbourhood. The IMD scores are then ranked nationally. In this study, the national ranking was divided into quintiles with the first quintile being the least deprived and the fifth, the most. Each woman is linked by her postcode at the time of birth to one of these neighbourhoods and is, therefore, assigned a deprivation quintile (see online supplemental methods section for more details)

10.1136/jech-2024-222060.supp1 Supplementary data

The second exposure was ethnicity, defined using the Office for National Statistics categorisation system^13^ collapsed into eight groups based on the categories used for theMothers and Babies: Reducing Risk through Audits and Confidential Enquiries across the UK (MBRRACE-UK) perinatal mortality surveillance report.^8^

The outcome was defined as the English Maternal Morbidity Outcome Indicator (EMMOI).^14^ This is a composite outcome consisting of 17 diagnoses and 9 procedures, which can be used as a single measure of SMM during pregnancy or childbirth using data from HES APC. The list of the relevant diagnoses/procedures and their codes are included in online supplemental table S1.

A Directed Acyclic Graph was used to conceptually represent which variables were confounders, colliders, effect modifiers or mediators based on both existing literature and clinical knowledge *a priori*. Based on the existing literature,^3^ maternal age at childbirth was deemed to be potential effect modifier *a priori*, ethnicity was examined as an effect modifier in the relationship between IMD and SMM, and IMD was examined as an effect modifier in the relationship between ethnicity and SMM.

### Statistical analysis

Statistical analysis was performed using Stata V.17. Statistical significance was assumed to be a p value of less than 0.05.

### Descriptive statistics

The incidence of SMM at the time of birth over the whole study population and in each IMD quintile and ethnic group was calculated using the number of maternities (women with either live or stillbirths) as the denominator. The characteristics of the women in the study population are presented as number and percentage in each group stratified by IMD quintile.

### Univariable analysis

The baseline group (the reference category) was the least deprived quintile for deprivation and the white British group was the baseline group for ethnicity. Logistic regression was used to estimate the ORs of SMM and their 95% CIs in each of the IMD quintiles and ethnic groups. Likelihood ratio testing was used, comparing a model with IMD as a nominal variable to a model with IMD as an ordinal variable, to test for departure from linearity.

### Multivariable analysis

Three models were built using multivariable logistic regression to estimate the ORs of SMM and their 95% CIs in each of the IMD quintiles and ethnic groups, using a complete case analysis. Model 1 included IMD, age and ethnicity. Likelihood ratio testing for linearity was used to determine whether age and the IMD quintiles should be modelled, respectively, as continuous and ordinal variables or as categorical variables. Model 2 included additional adjustment for the medical and psychological factors including prior history of pre-exisiting medical conditions and mental illness. Model 3 included additional adjustment for the health behaviour factors obesity, smoking and substance misuse. Likelihood ratio testing was performed to determine whether there was an interaction between age category and IMD or between ethnicity and IMD. There was no evidence of an interaction; therefore, the results were not stratified. Average adjusted predictions for each IMD quintile and ethnic group were then calculated.

### Sensitivity analyses

Sensitivity analyses were undertaken to assess the impact of missing data, first by excluding all women with missing information on parity in the HES APC childbirth record; second, by imputing the values for maternal characteristics if they were missing in the confounding covariates in the multivariable analysis, using fully conditional specification multiple imputation by chained equations to generate 50 datasets and pooling estimates using Rubin’s rules.^15^ E-values were also calculated to quantify the strength of any unmeasured confounding needed to negate the observed results.

## Results

### Characteristics of the study population

The characteristics of the women stratified by IMD quintile and ethnicity are shown in tables 1 and 2. For the final study population of 1 178 756 women (online supplemental figure S2), the overall risk of SMM at the time of childbirth was 1.63%. The characteristics of the women who had SMM stratified by IMD and ethnicity are shown in online supplemental tables S3 and S4. The breakdown of the number of SMM events by each diagnosis and procedure are shown in online supplemental table S5.

**Table 1:** Characteristics of women stratified by IMD quintile (most to least deprived) N (%)

	IMD quintile				
	Most deprived 20%	More deprived 20–40%	Less deprived 40–60%	Less deprived 60–80%	Least deprived 80–100%
Ethnicity					
White	173 117 (64.0%)	182 043 (68.9%)	180 926 (75.4%)	173 348 (79.3%)	150 829 (81.4%)
Black or Black British—Caribbean	3338 (1.2%)	2382 (0.9%)	1252 (0.5%)	727 (0.3%)	372 (0.2%)
Black or Black British—African	10 814 (4.0%)	6834 (2.6%)	3226 (1.3%)	1947 (0.9%)	1170 (0.6%)
Asian or Asian British—Indian	7221 (2.7%)	9826 (3.7%)	7838 (3.3%)	5864 (2.7%)	4808 (2.6%)
Asian or Asian British—Pakistani	16 441 (6.1%)	8359 (3.2%)	4186 (1.7%)	2485 (1.1%)	1556 (0.8%)
Asian or Asian British—Bangladeshi	5479 (2.0%)	2915 (1.1%)	1202 (0.5%)	682 (0.3%)	434 (0.2%)
Mixed	6388 (2.4%)	5400 (2.0%)	4032 (1.7%)	3280 (1.5%)	2411 (1.3%)
Other	26 215 (9.7%)	24 518 (9.3%)	17 741 (7.4%)	12 778 (5.8%)	9470 (5.1%)
Missing	21 484 (7.9%)	22 028 (8.3%)	19 575 (8.2%)	17 591 (8.0%)	14 224 (7.7%)
Age group					
<20	30 786 (11.4%)	16 762 (6.3%)	10 799 (4.5%)	7038 (3.2%)	4166 (2.2%)
20–25	77 849 (28.8%)	56 374 (21.3%)	40 830 (17.0%)	29 920 (13.7%)	19 646 (10.6%)
25–30	81 861 (30.3%)	82 302 (31.1%)	74 489 (31.0%)	67 410 (30.8%)	54 174 (29.2%)
30–35	54 598 (20.2%)	73 373 (27.8%)	76 629 (31.9%)	77 509 (35.4%)	72 455 (39.1%)
35–40	20 911 (7.7%)	29 686 (11.2%)	31 247 (13.0%)	30 711 (14.0%)	29 125 (15.7%)
>40	4492 (1.7%)	5808 (2.2%)	5984 (2.5%)	6114 (2.8%)	5708 (3.1%)
Pre-existing medical conditions*	28 449 (10.5%)	26 811 (10.1%)	25 560 (10.7%)	23 852 (10.9%)	20 358 (11.0%)
Pre-existing mental health condition*	14 677 (5.4%)	11 805 (4.5%)	9909 (4.1%)	8481 (3.9%)	6710 (3.6%)
Obesity/ overweight*	64 854 (24.0%)	54 925 (20.8%)	46 605 (19.4%)	40 468 (18.5%)	31 083 (16.8%)
Substance misuse or smoking*	33 480 (12.4%)	27 036 (10.2%)	22 613 (9.4%)	19 345 (8.8%)	14 371 (7.8%)
Total	**270 497 (22.9%)**	**264 305 (22.4%)**	**239 978 (20.4%)**	**218 702 (18.6%)**	**185 274 (15.7%)**

*See list of conditions included list in online supplemental table S2.

IMD, Index of Multiple Deprivation.

**Table 2:** Characteristics of women stratified by ethnicity N (%)

	Ethnicity							
	White	Black or Black British—Caribbean	Black or Black British—African	Asian or Asian British—Indian	Asian or Asian British—Pakistani	Asian or Asian British—Bangladeshi	Mixed	Other
IMD quintile								
Most deprived 20%	173 117 (20.1%)	3338 (41.4%)	10 814 (45.1%)	7221 (20.3%)	16 441 (49.8%)	5479 (51.1%)	6388 (29.7%)	26 215 (28.9%)
More deprived 20–40%	182 043 (21.2%)	2382 (29.5%)	6834 (28.5%)	9826 (27.6%)	8359 (25.3%)	2915 (27.2%)	5400 (25.1%)	24 518 (27.0%)
Less deprived 40–60%	180 926 (21.0%)	1252 (15.5%)	3226 (13.4%)	7838 (22.0%)	4186 (12.7%)	1202 (11.2%)	4032 (18.7%)	17 741 (19.6%)
Less deprived 60–80%	173 348 (20.2%)	727 (9.0%)	1947 (8.1%)	5864 (16.5%)	2485 (7.5%)	682 (6.4%)	3280 (15.2%)	12 778 (14.1%)
Least deprived 80–100%	150 829 (17.5%)	372 (4.6%)	1170 (4.9%)	4808 (13.5%)	1556 (4.7%)	434 (4.1%)	2411 (11.2%)	9470 (10.4%)
Age group								
<20	58 755 (6.8%)	688 (8.5%)	736 (3.1%)	213 (0.6%)	751 (2.3%)	222 (2.1%)	1799 (8.4%)	3054 (3.4%)
20–25	169 709 (19.7%)	2129 (26.4%)	4482 (18.7%)	3304 (9.3%)	8615 (26.1%)	2895 (27.0%)	4553 (21.2%)	14 362 (15.8%)
25–30	256 361 (29.8%)	2297 (28.5%)	8664 (36.1%)	12 100 (34.0%)	13 992 (42.4%)	4736 (44.2%)	5881 (27.3%)	27 364 (30.2%)
30–35	255 669 (29.7%)	1749 (21.7%)	6580 (27.4%)	14 405 (40.5%)	6987 (21.2%)	2177 (20.3%)	6018 (28.0%)	29 198 (32.2%)
35–40	100 368 (11.7%)	932 (11.5%)	2797 (11.7%)	4685 (13.2%)	2199 (6.7%)	561 (5.2%)	2728 (12.7%)	13 778 (15.2%)
>40	19 401 (2.3%)	276 (3.4%)	732 (3.1%)	850 (2.4%)	483 (1.5%)	121 (1.1%)	532 (2.5%)	2966 (3.3%)
Pre-existing medical conditions*	106 525 (12.4%)	1155 (14.3%)	1616 (6.7%)	2437 (6.9%)	2664 (8.1%)	747 (7.0%)	2312 (10.7%)	5525 (6.1%)
Pre-existing mental health condition*	46 133 (5.4%)	386 (4.8%)	413 (1.7%)	463 (1.3%)	737 (2.2%)	235 (2.2%)	898 (4.2%)	1649 (1.8%)
Obesity/overweight*	185 452 (21.5%)	2163 (26.8%)	6213 (25.9%)	4776 (13.4%)	6911 (20.9%)	1902 (17.7%)	4330 (20.1%)	12 909 (14.2%)
Substance misuse or smoking*	104 395 (12.1%)	792 (9.8%)	709 (3.0%)	1044 (2.9%)	1450 (4.4%)	430 (4.0%)	2325 (10.8%)	3807 (4.2%)
Total	**860 263 (79.4%)**	**8071 (0.7%)**	**23 991 (2.2%)**	**35 557 (3.3%)**	**33 027 (3.0%)**	**10 712 (1.0%)**	**21 511 (2.0%)**	**90 722 (8.4%)**

*See list of conditions included list in online supplemental table S2.

IMD, Index of Multiple Deprivation.

### Univariable analysis

There was no evidence of departure from linearity (p=0.623) between deprivation quintile and SMM at the time of birth. Compared with the least deprived quintile, the odds of SMM at the time of childbirth were 6% (95% CI 1% to 12%) more for the second to least deprived quintile, 10% (95% CI 5% to 15%) more for the third most deprived quintile, 15% (95% CI 9% to 20%) more for the fourth most deprived and 20% (95% CI 14% to 25%) more for the most deprived quintile. Being from a black or black British African ethnic group had a 1.89 times (95% CI 1.74 to 2.04), black or black British Caribbean 1.73 (95% CI 1.51 to 1.99), Asian or Asian British Indian 1.23 (95% CI 1.13 to 1.33), Asian or Asian British Pakistani 1.47 (95% CI 1.37 to 1.59), Asian or Asian British Bangladeshi 1.64 (95% CI 1.45 to 1.85), Mixed 1.22 (95% CI 1.10 to 1.35) and Other 1.37 (95% CI 1.31 to 1.44) greater odds of SMM compared with being from a white ethnicity. The results of the univariable analysis of IMD, ethnicity and the other covariates are shown in table 3.

**Table 3:** Multivariable models to show associations with SMM

	Unadjusted model	Model 1*	Model 2†	Model 3‡
	OR (95% CI)	OR (95% CI)	OR (95% CI)	OR (95% CI)
IMD quintile				
Most deprived 20%	1.20 (1.14 to 1.25)	1.16 (1.11 to 1.23)	1.16 (1.10 to 1.22)	1.13 (1.07 to 1.19)
More deprived 20–40%	1.15 (1.09 to 1.20)	1.12 (1.06 to 1.17)	1.11 (1.06 to 1.17)	1.10 (1.04 to 1.15)
Less deprived 40–60%	1.10 (1.05 to 1.15)	1.09 (1.03 to 1.15)	1.09 (1.03 to 1.14)	1.08 (1.02 to 1.13)
Less deprived 60–80%	1.06 (1.01 to 1.12)	1.07 (1.02 to 1.13)	1.07 (1.01 to 1.13)	1.06 (1.01 to 1.12)
Least deprived 80–100%	1(ref)	1(ref)	1(ref)	1(ref)
Ethnicity				
White	1(ref)	1(ref)	1(ref)	1(ref)
Black or Black British—Caribbean	1.73 (1.51 to 1.99)	1.69 (1.47 to 1.94)	1.68 (1.46 to 1.93)	1.66 (1.44 to 1.90)
Black or Black British—African	1.89 (1.74 to 2.04)	1.81 (1.67 to 1.96)	1.86 (1.72 to 2.02)	1.84 (1.70 to 2.00)
Asian or Asian British—Indian	1.23 (1.13 to 1.33)	1.19 (1.10 to 1.29)	1.23 (1.13 to 1.33)	1.26 (1.16 to 1.36)
Asian or Asian British—Pakistani	1.47 (1.37 to 1.59)	1.44 (1.33 to 1.55)	1.47 (1.36 to 1.59)	1.49 (1.38 to 1.61)
Asian or Asian British—Bangladeshi	1.64 (1.45 to 1.85)	1.60 (1.41 to 1.81)	1.64 (1.45 to 1.86)	1.68 (1.48 to 1.90)
Mixed	1.22 (1.10 to 1.35)	1.21 (1.09 to 1.33)	1.22 (1.10 to 1.35)	1.23 (1.11 to 1.36)
Other	1.37 (1.31 to 1.44)	1.33 (1.27 to 1.40)	1.37 (1.31 to 1.44)	1.41 (1.34 to 1.48)
Age group				
<20	1.20 (1.11 to 1.29)	0.85 (0.79 to 0.92)	0.86 (0.80 to 0.93)	0.87 (0.81 to 0.94)
20–25	1 to (ref)	1(ref)	1(ref)	1(ref)
25–30	1.24 (1.15 to 1.33)	1.04 (0.99 to 1.08)	1.04 (0.99 to 1.08)	1.04 (1.00 to 1.09)
30–35	1.27 (1.18 to 1.36)	1.09 (1.04 to 1.14)	1.09 (1.05 to 1.14)	1.11 (1.06 to 1.16)
35–40	1.37 (1.27 to 1.48)	1.18 (1.12 to 1.25)	1.17 (1.11 to 1.24)	1.20 (1.13 to 1.26)
>40	1.72 (1.55 to 1.91)	1.51 (1.38 to 1.65)	1.48 (1.35 to 1.62)	1.51 (1.38 to 1.65)
Pre-existing medical conditions§	1.38 (1.33 to 1.44)		1.39 (1.33 to 1.45)	1.35 (1.29 to 1.41)
Pre-existing mental health diagnosis§	1.24 (1.16 to 1.32)		1.17 (1.10 to 1.25)	1.15 (1.08 to 1.24)
Substance misuse or smoking§	1.06 (1.01 to 1.11)			0.99 (0.94 to 1.04)
Obesity/overweight§	1.42 (1.37 to 1.46)			1.40 (1.35 to 1.45)

*Model 1 additional adjustment for age (in 5-year categories) and ethnicity (in eight categories).

†Model 2 additional adjustment for medical and psychological factors including pre-exisiting medical conditions and pre-exisiting mental health problems.

‡Model 3 additional adjustment health behaviours, obesity, smoking and substance misuse.

§See list of conditions included list in online supplemental table S2.

IMD, Index of Multiple Deprivation; SMM, severe maternal morbidity.

### Multivariable analysis

**Figure 1:**
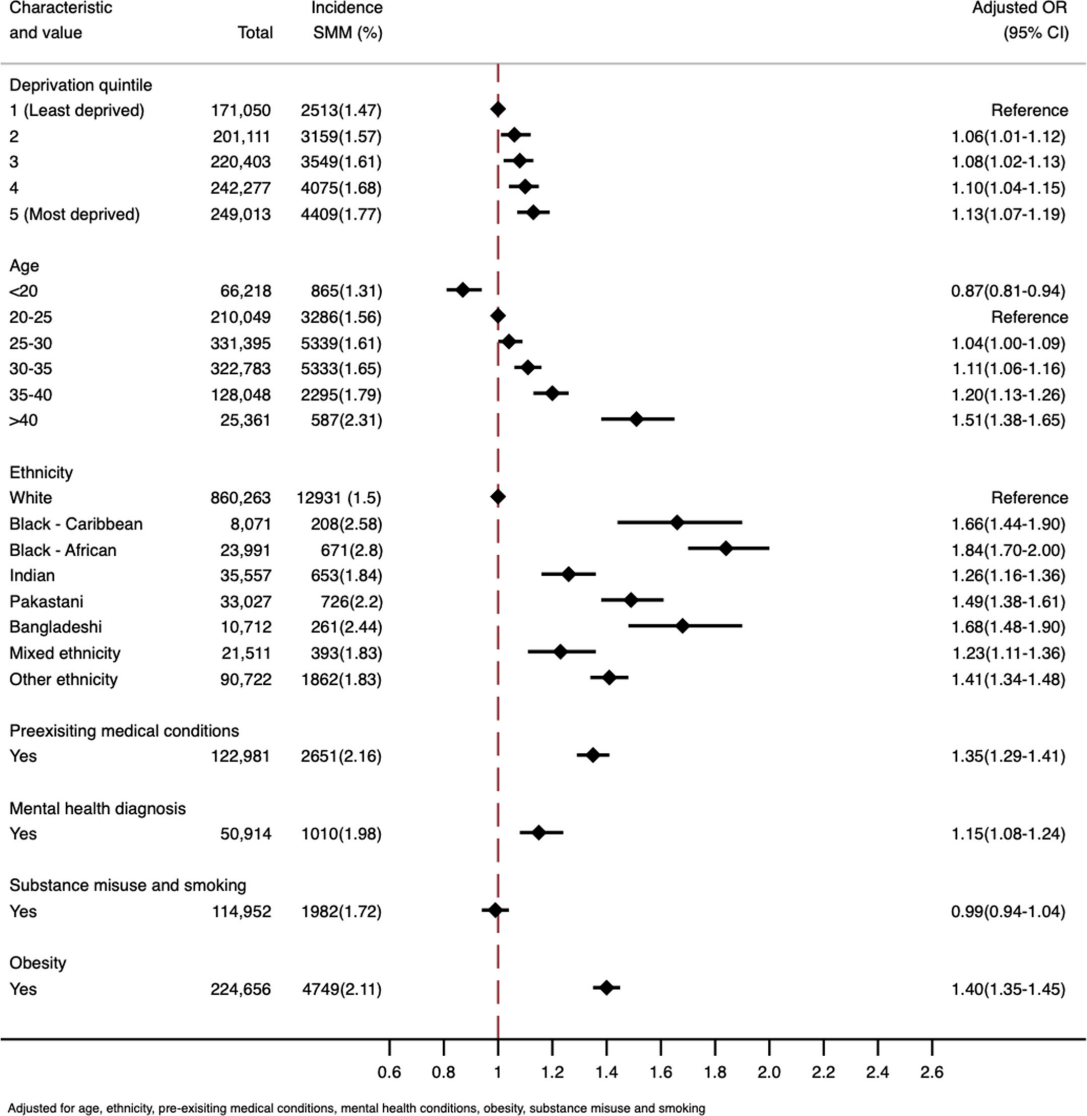
The fully adjusted variables from model 3. SMM, severe maternal morbidity.

The ORs and their 95% Cis for the sequential models are shown in table 3 and the ORs from the fully adjusted model are shown in figure 1. Age was modelled as a categorical variable as there was statistically significant evidence of departure from linearity (p<0.001). After adjustment for all confounding factors (table 3, model 3), there was a linear trend (p=0.001) with a 3% (95% CI 1% to 4%) increase in the odds of SMM for every deprivation quintile as deprivation increased. Compared with the least deprived quintile, the odds of SMM at the time of childbirth were 6% (95% CI 1% to 12%) more for the second to least deprived quintile, 8% (95% CI 2% to 13%) more for the third most deprived quintile, 10% (95% CI 4% to 15%) more for the fourth most deprived and 13% (95% CI 7% to 19%) more for the most deprived quintile. There was no significant interaction between IMD and age (p=0.864) or IMD and ethnicity (p=0.486). All minoritised ethnic groups had a significantly raised odds of an SMM outcome compared with white women in the fully adjusted model. The results of the average adjusted predictions are shown in online supplemental table S6.

### Sensitivity analysis

The ORs and their 95% CIs from the sensitivity analysis are shown in online supplemental table S7. The ORs did not differ materially after multiple imputation or after excluding the women with parity coded as missing. The e-value results are shown in online supplemental table S8.

## Discussion

### Main findings

This study contributes to the existing body of research exploring risk factors for SMM in high-income countries. The findings showed a linear relationship between neighbourhood deprivation and SMM at the time of birth, in primiparous women in England. Minoritised ethnic groups also experienced greater odds of SMM, with black of black British African having nearly two times the odds compared with white women. No ethnic group appeared to be more affected by the higher rates of SMM associated with deprivation, and the both deprivation and ethnicity appear to have independent associations with SMM rather than one being a surrogate marker for the other.

### Strengths and limitations

The strengths and limitations of using the HES APC for research purposes are discussed in online supplemental material.

Using a composite outcome helps avoid the problem of individual severe morbidities being relatively rare as well as conditions not being correctly coded in routinely collected health data, leading to false negatives. Although the EMMOI captures a range of outcomes that do not all share a direct a causal pathway, it is useful marker both for the overall preconception and pregnancy health and for the quality of care given during pregnancy and birth.

However, a key limitation of this study is the misclassification of the exposure. The association between deprived individuals and their risk of SMM may be attenuated as not everyone who lives in a deprived area is individually deprived.^16^ In addition, there are also some important missing dimensions to the IMD index such as social well-being and environmental quality,^17^ and, thus, it is not possible in this study to fully elucidate causal pathways that link deprivation to maternal morbidity without incorporating such factors.^18^

Additionally, some of the confounding factors included in the multivariable regression analysis may also be on the causal pathway. The confounding factors of pre-exisiting medical conditions and mental health history are individual factors which are included in calculating the IMD score of each area. There is, therefore, a risk of over adjustment, as the adjustments include both potential mediators and individual markers of deprivation. Thus, the results of the association between the IMD and SMM in the above-adjusted models need to be interpreted taking these factors into consideration.^19^

### Interpretation (in light of other evidence)

To our knowledge this is the first study to look at the relationship between neighbourhood deprivation and a composite measure of SMM in England. However, this association has been shown similar high-income countries including Canada,^3^ Australia^5^ and New Zealand.^20^ There are many possible reasons for the higher rate of SMM in more deprived neighbourhoods and the relationship between the confounding factors and causal pathways are complex. For example, having a pre-existing medical condition can be both a cause and consequence of living in a deprived area. It has been hypothesised that the increased risk of SMM for the most deprived women could be driven by a difference in individual health behaviours such as smoking and substance misuse, psychosocial factors such as chronic stress, material factors such as low income affecting the quality of nutrition and obesity, and environmental factors such as air pollution and poor housing.^3 5 18 20 21^ Indeed, in this study, it appears that some of the individual or ‘compositional’ disadvantage factors, which affect prepregnancy health, partially account for the relationship between deprivation and SMM, as the association is reduced after adjusting for individual pre-existing medical and mental health conditions, smoking, substance use and obesity. One explanation for the remaining risk after accounting for these individual factors is the contextual effects of living in a deprived area beyond the risks of being individually disadvantaged. It could also be explained by differences between care quality and access between different social groups. In the UK, secondary analysis of a National Maternity Survey^22^ showed that women who lived in the most deprived IMD quintile were less likely to have antenatal care and were more likely to report being treated disrespectfully or spoken to in a way they could not understand by doctors. It is possible that this difference in treatment of women by healthcare providers and access to maternity services may contribute to the differences in SMM for women living in the most deprived areas in England.

Studies conducted both in the UK and similar high-income countries have demonstrated that being from a minoritised ethnic group is a risk factor for maternal morbidity,^6 7^ which is also seen in our study. Indeed, being of a minoritised ethnicity appeared to have the greatest impact on risk of SMM compared with the other risk factors. One hypothesis previously suggested for what could be driving this trend is that ethnicity may be a surrogate marker for socioeconomic deprivation.^23^ However, a case–control study in the UK showed that the risk of morbidity is 43%–83% higher in women from a minoritised ethnic group compared with white women, and this was not confounded by occupation, which was used as a marker for socioeconomic status.^24^ This, alongside the results of our study, suggests that socioeconomic status (or deprivation) and ethnicity appear to be independently associated with increasing the risk of maternal morbidity, rather than one being mostly a marker for the other.

It has been argued^25^ that systemic racism, rather than genetic or biological phenomena, drives the increased risk in childbirth for black, brown and mixed ethnicity women. Racism can affect health outcomes throughout the life course, in multiple forms and through multiple upstream, midstream and downstream pathways.^26^ However, in this study, the increased risk of SMM in women from a minoritised ethnic group is not strongly related to the medical, behavioural or psychological factors, which were included and adjusted for in this study. A report reviewed in-depth testimony from over 300 people and found that women from minoritised ethnicities felt unsafe, were ignored and disbelieved, were subjected to racism by caregivers, were not given a proper choice or the means to give informed consent, reported being subjected to coercion, regularly dehumanised and were disproportionately affected by structural barriers to care. This report, alongside the results from our study, suggests that coexisting medical problems or health behaviours, which were able to be captured by the methods of this study, do not appear to account for the increased risk in childbirth for women from minoritised ethnic groups.

Finally, we were only able to adjust for pre-existing health conditions diagnosed before the time of birth. The long-term effects of socioeconomic deprivation and systemic racism may not have manifested in the form of chronic disease diagnosis at this point in the life course. However, these long-term effects may be an important underlying contributing factor to poorer preconception and pregnancy health and therefore risk of SMM.^27^

## Conclusion

This study has shown that socioeconomic deprivation and ethnicity are important factors associated with SMM. The increased risk associated with being from a minoritised ethnic group cannot be accounted for simply by living in a deprived area, or the presence of a pre-existing physical or mental health conditions, differences in age or behavioural factors captured in this database. This suggests that other factors are driving this increased risk, for example, those relating to care or the chronic effects of disadvantage, which have not yet manifested in disease. This study adds to the increasing body of evidence, that alongside the development of targeted and evidence-based interventions, there is an urgent need to address the underlying systems and structures which are driving the increased risk for disadvantaged groups.

## Data Availability

Data may be obtained from a third party and are not publicly available. The data extract was derived from the English National Hospital Episode Statistics Admitted Patient Care (HES-APC) database with linkage to national mortality civil registrations (https://digital.nhs.uk/data-and-information/data-tools-and-services/dataservices/linked-hes-ons-mortality-data). Linked HES-APC and mortality data are available upon application to NHS England (formerly NHS Digital).
