# Supplementary material for: Associations between neighbourhood deprivation, ethnicity and maternal health outcomes in England: a nationwide cohort study using routinely collected healthcare data
Source: J Epidemiol Community Health. 2024 Jun 4;78(8):500–7. doi: 10.1136/jech-2024-222060 (PMC11287519; doi:10.1136/jech-2024-222060)
Supplement: Supplementary data [file jech-2024-222060supp001.pdf]

Supplementary material

Supplementary Methods

Study Population

This study included all primiparous women aged 10-54 who gave birth between 1st January 2016 and 31st December 2021, in hospital, with a gestational age at childbirth of greater than 20 weeks. All NHS trusts in England were included. Homebirths, which account for around 2.3% of births[1] in England, were not covered by the dataset. A 'look back'[2] method was used to determine parity. Women were categorised as primiparous if it was the index birth in the study time period, there was no birth codes **(Table S1)** in the time period from 2003 to the index birth and parity was coded as zero or missing in the pregnancy record. The study was restricted to primiparous women to avoid clustering of outcomes within mothers who may have had more than one birth in the study period.

Exposures

The first exposure was defined based on the 2019 release of the IMD[3]. The IMD is a composite area-based deprivation measure comprising seven domains: income, education, skills and training, employment, health and disability, crime, living environment and barriers to housing and services. England is divided into 32,844 areas, known as Lower Super Output Areas (LSOAs) of around 1500 residents, and each LSOA is assigned a score and a rank for the individual domains of deprivation. A weighted sum of the ranks for each domain is used to calculate an overall IMD score. The IMD scores are then ranked nationally. In this study, the national ranking was divided into quintiles with the first quintile being the least deprived and the fifth, the most. The maternal residential postcode at the time of birth was used to

Geddes-Barton D, *et al. J Epidemiol Community Health* 2024; 78:500–507. doi: 10.1136/jech-2024-222060

assign each woman into an LSOA and an IMD quintile. The composite IMD score captures both compositional socioeconomic disadvantages, for example the number of people living in an area on unemployment benefits, and contextual deprivation, for example living in an area with poor access to services or high air pollution.

The second exposure was ethnicity, defined using the Office for National Statistics categorisation system[4] collapsed into eight groups based on the categories used for the MBRRACE-UK perinatal mortality surveillance report[5]: White, Black or Black British African, Black or Black British Caribbean, Asian or Asian British Indian, Asian or Asian British Pakistani, Asian or Asian British Bangladeshi, Mixed and Other. The Other category consisted of women from any other Black or Asian background, Chinese or any other ethnic group. The Office for National Statistics made person level comparisons between ethnicity coded in HES and self-reported ethnicity in the 2011 census (considered gold standard). Out of the 66% of the total population recorded in the 2011 census which could both be linked with HES and had their ethnicity recorded, there was 90.7% agreement when ethnicity was divided into 18 ethnic categories and 95.8% agreement when it was divided into 5 ethnic categories between the two data sources.

Confounders

Confounding factors included the following measured at the individual level: maternal age at childbirth, obesity, pre-existing medical conditions, pre-existing mental health conditions, substance misuse and smoking. Ethnicity and IMD were also adjusted for each other in the multivariable analysis. Maternal age at childbirth was grouped into five-year categories except for ages younger than 20 or older than 40+ which were grouped into single categories. Information on the following potential confounding factors was obtained on the basis of ICD-

Geddes-Barton D, *et al. J Epidemiol Community Health* 2024; 78:500–507. doi: 10.1136/jech-2024-222060

10 codes: obesity or overweight (yes/no), history of pre-existing medical condition (yes/no), history of pre-existing mental health problems (yes/no), substance misuse and smoking (yes/no). Women were assumed not to have the above confounding factors if they were not coded in any of the hospital records from the preceding length of time listed in **Table S2** to the start of the index pregnancy. The list of relevant pre-existing medical conditions and mental health problems and the codes for all confounding variables is provided in **Table S2**.

Outcome

The outcome was defined as the English Maternal Morbidity Outcome Indicator (EMMOI)[6]. This is a composite outcome that includes 17 diagnoses and 9 procedures, adapted from the Australian Maternal Morbidity Outcome Indicator[7] in 2016, which can be used as a single measure of severe morbidity during pregnancy or childbirth using data from HES APC. The list of the relevant diagnoses/ procedures and their codes are included in **Table S1**. SMM was coded as a dichotomous variable, with a woman being given the score of 1 if she had one of more of these codes during her birth episode or 0 if she did not.

Interactions

Based on existing literature,[8] maternal age at childbirth was deemed to be potential effect modifier *a priori*, ethnicity was examined as an effect modifier in the relationship between IMD and severe maternal morbidity, and IMD was examined as an effect modifier in the relationship between ethnicity and severe maternal morbidity.

Geddes-Barton D, *et al. J Epidemiol Community Health* 2024; 78:500–507. doi: 10.1136/jech-2024-222060

Strengths and limitations of HES APC

The key strength of this data source is its universal coverage of all NHS hospital births in England, allowing for a nationwide population study. This reduces selection bias as information on 97% of all births in England are reported in HES APC. Furthermore, the HES APC is administrative data collected for NHS payment purposes. It is therefore easily obtained without need for resource-intensive bespoke collection. It contains both time stamped clinical information as well as demographic variables such as the IMD and ethnicity. However, as the data is not collected prospectively for the purpose of this research question it falls short on key variables such useful information on individual socio-economic factors. Furthermore, the clinical coders rely on the quality and detail of discharge summaries which are created by busy clinical staff. Thus, there is a risk of conditions not being coded for, creating false negatives and misclassification if these data are used for research purposes. Notably in this study, the prevalence of overweight and obese women, and women with pre-existing physical or mental health conditions are low compared to other national sources[9]. Furthermore, every birth record contains optional additional maternity data, not mandated for collection. This leads to large variations in data quality and completeness between hospitals.[10] Parity is been shown to be unreliably coded in the maternity data with only 59% or trusts having an expected distribution of parity. However, this study used the ‘look back’ method with eighteen years of data to reclassify multiparous women with a previous delivery code who may have been incorrectly classified as primiparous, and to determine parity in those women for whom parity had not been recorded.

Figure S1. Directed Acyclic Graph (DAGs). *The DAGs only included the variables which were used in the multivariable analysis and were available in the HES APC.

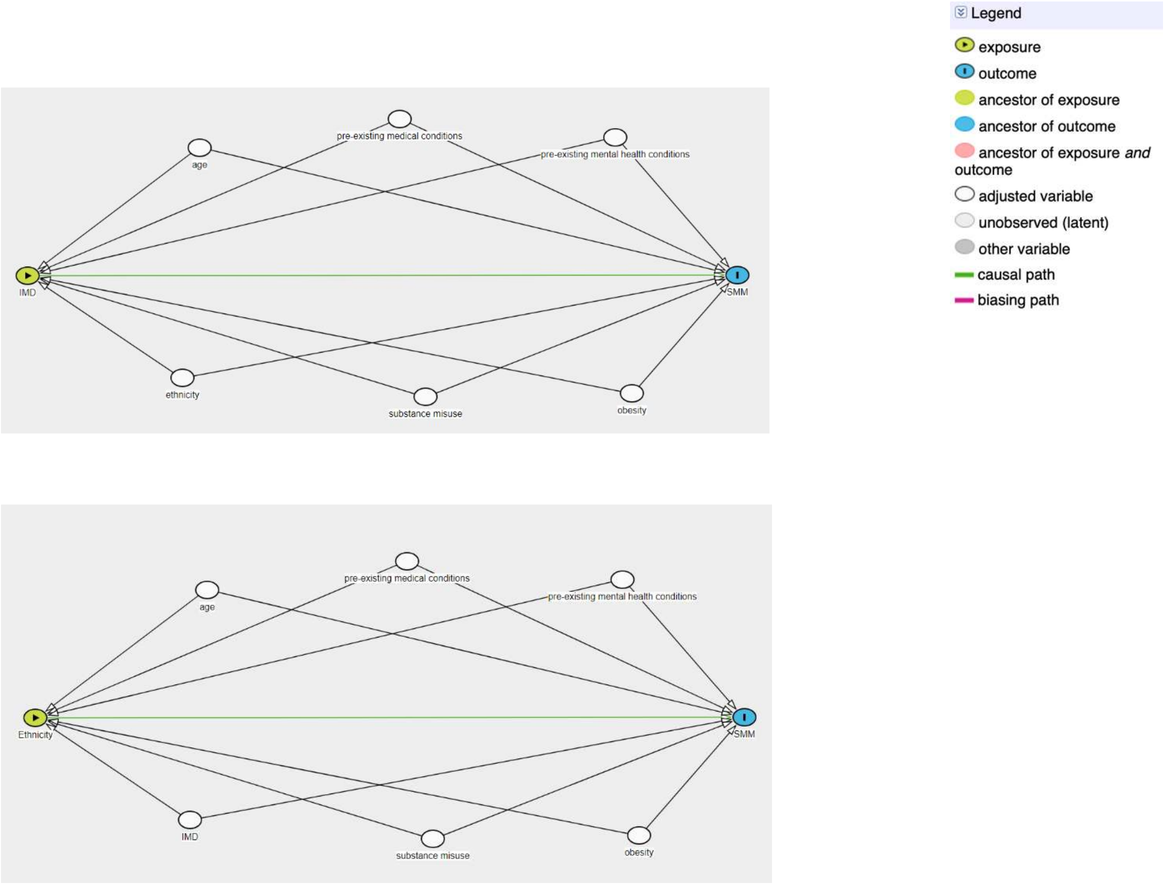

Figure S2: Identification of the study population of included primiparous women

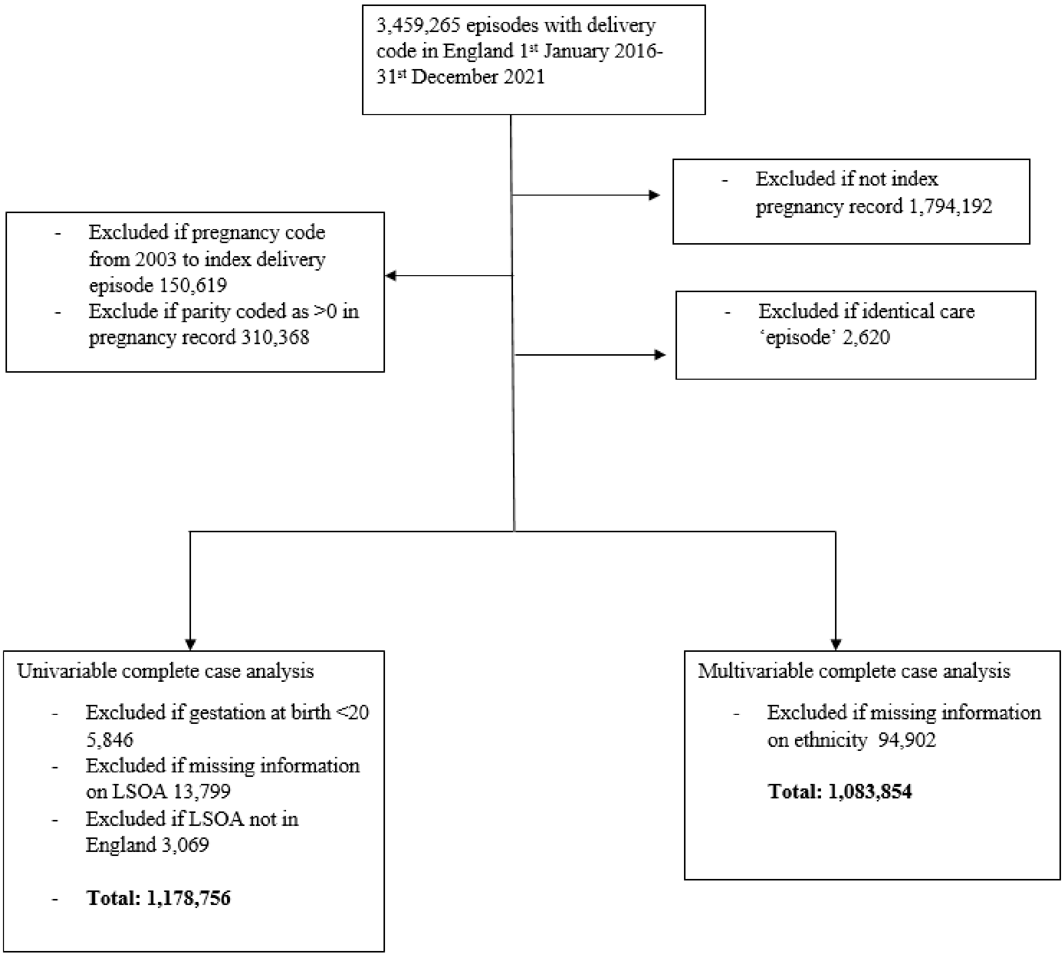

Table S1. **Code list English Maternal Morbidity Outcome Indicator (EMMOI) coded for at the hospital birth episode**

**Morbidity Event**	**Code**
**Diagnosis**	**ICD10**
Acute abdomen	K35, K37, K65.0, K65.9, N73.3, N73.5
Acute renal failure	O90.4, N17, N19, N99.0
Acute psychosis	F23, F53.1
Cardiac arrest/ failure or infarction	O89.1, O74.2, O90.3, I21, I42, I43, I46, I50, J81
Cerebral oedema/coma	G93.6, R40.2
Disseminated Intravascular Coagulation	D65
Cerebrovascular accident	I60, I61, I62, I63, I64
Major complications of anaesthesia	O74.0, O74.1, O74.2, O74.3, O74.9, O89.0, O89.1, O89.2, O29.0, O29.1, O29.2
Obstetric Embolism (including Amniotic embolism)	O88 (O88.0, O88.1, O88.2, O88.3, O88.8)
Shock	R57.0, R57.1, R57.2, R57.8, R57.9, O75.1, T80.5, T88.6
Sickle cell anaemia with crisis	D57.0
Status asthmaticus	J46
Status epilepticus	G41, G41.0, G41.1, G41.2, G41.8, G41.9
Uterine rupture	O71.0, O71.1
Eclampsia	O15.0, O15.1, O15.2, O15.9
Sepsis	O85
Cerebral Venous Thrombosis	O87.3
Procedures	**OPCS 4.7 codes**
Assisted ventilation including tracheostomy	E85.1, E85.2, E42.1, E42.2, E42.3, E42.8, E42.9
Curettage in combination with a general anaesthetic	R28.1 + Y80
Dialysis	X40, X41.1, X42.1
Evacuation of haematoma	P09.3, P27.1, T34.1, T34.2, T34.3, T45.1, T45.2, T45.3, T45.4, Y22.1
Hysterectomy	Q07.1, Q07.2, Q07.3, Q07.4, Q07.5, Q08
Procedures to reduce blood flow to uterus	L69.3, L69.4, L70.2, L70.3, L71.3, L93.3, L94.1, L94.6, L94.7, L99.1, L99.5, L99.6
Re-closure of disrupted caesarean section wound	T28.3, T30.1, T30.2, T30.3, T30.4, S42.3, S42.4, S60.4
Repair of bladder or cystostomy	M37.2, M37.3, M37.5, M37.8, M37.9, M38.2, M38.3, M73.6, M73.7
Repair of intestine	G58, G69, G70, G78, H06, H07, H08, H09, H10, H11, H23, H26, H29, H33, T37.4, T38.4, T42.1

Table S2. ICD-10 codes for co-variates

**Pre-existing Medical Condition*[1]**	**Code**	**Timing**
Diabetes	G590 G632 H280 H360 M142 N083 O240 O241 O243 H350 H352 E103 E113 E123 E133 E143 E10-E14	Any code from 2003 to start of pregnancy
Heart disease - ischaemic	I252 I20 I21 I22 I23 I24 I25 I270 I272	Any code from 2003 to start of pregnancy
Heart disease – heart failure and cardiomyopathy	I43 I50 I130 I110 I132	Any code from 2003 to start of pregnancy
Heart disease- congenital	Q20-Q26	Any code from 2003 to start of pregnancy
Heart disease – valve	I34<br>I35<br>I05-I08	Any code from 2003 to start of pregnancy
Hypertension	I10-I13 I15	Any code from 2003 to start of pregnancy
Thyroid disease	E035 E038 E039 E050 E051 E052 E055 E058 E059 E062 E063 E065 E069 H062	Any code from 2003 to start of pregnancy
Obstructive lung disorders – cystic fibrosis	E84	Any code from 2003 to start of pregnancy
Restrictive lung disorders	J60 J61 J62 J63 J64 J65 J66 J67 J84 J841 J701 J703 J704 G532 M633 D86	Any code from 2003 to start of pregnancy
Polyarthropothies	I730 J990 M350 M05 M06 M34 L93 M32	Any code from 2003 to start of pregnancy
Obstructive lung disorders	J45, J46	Any code from 2003 to start of pregnancy
Inflammatory bowel disease	K51 K50	Any code from 2003 to start of pregnancy
Coeliac	K900	Any code from 2003 to start of pregnancy
Infective Hepatitis	B150 B160 B190 B18	Any code from 2003 to start of pregnancy
Alcohol related liver disease	K701 K702 K703 K704 K70	Any code from 2003 to start of pregnancy
Cirrhosis and liver failure	K717 K740 K741 K742 K744 K745 K746 K72	Any code from 2003 to start of pregnancy
Other liver disease	K743 K754 K711 K762 K763	Any code from 2003 to start of pregnancy
Cerebrovascular disease	I61 I64 I60 I65 I66 I691 I630 I631 I632 I633 I634 I635 I638 I639 I693 G463 G464 G465 G466 G467 G468 I694 I690 G450 G451 G452 G453 G454 G458 G459 G460 G461 G462	Any code from 2003 to start of pregnancy
Epilepsy	G40 G41	Any code from aged 11+ to start of pregnancy
Chronic kidney disease	N185 T824 Y602 Y612 Y841 Z491 Z492 Z992 N165 T861 Z940 N183 N184 N187 N188 N189 N186	Any code from 2003 to start of pregnancy

Psoriasis and Eczema	l20 l40 M090	Any code from aged 11+ to start of pregnancy
HIV	F024 B20 B21 B22 B23 B24 R75 Z21	Any code from 2003 to start of pregnancy
Cancer	C880 C882 C221 C751 C752 C753 C754 C755 C220 C222 C223 C224 C227 C229 C260C261 C268 C269 C300 C301 C380 C381 C382 C383 C384 C388 C390 C398 C399 C750 C758 C759 C883 C884 C887 C889 C797 C795 C784 C785 C793 C794 C787 C780 C781 C783 C788 C790 C791 C792 C796 C798 C799 C786 C782 C81 C91-C95 C82-C85 C86 C90 C40 C41 C70 – C72 C51-C53 C31-C34 C43-C49 C64-C67 C97 C00-C21 C23-C25 C37 C57 C58 C60 C63 C68 C69 C74 C76 C80 C96 C56 C73 C54 C55 C77 C50 D05	Any code from 2003 to start of pregnancy
Transplant	Z94	Any code from 2003 to start of pregnancy
Fibroids	D25	Any code from 2003 to start of pregnancy
Endometriosis	N80	Any code from 2003 to start of pregnancy
Inflammatory bowel syndrome	K58	Any code from 2003 to start of pregnancy
Post viral and related fatigue	G933	Any code from 2003 to start of pregnancy
Fibromyalgia	M797	Any code from 2003 to start of pregnancy
Thrombophilia and thromboembolic disease	D68, I74-I76, I26, I80-I82	Any code from 2003 to start of pregnancy
**Obesity**	E66, Z684	Any code
**Substance misuse[2]**	Coded from 5 years prior to index pregnancy up unto the start of pregnancy	
Mental and behavioural disorders due to psychoactive substance use	F11-F17, F19 (not F171)	
Finding of drugs not normally found in blood	R781-R785	
Poisoning by drugs, medicaments and biological substances	T36-T50 (not T506)	
Poisoning, undetermined intent	Y10-Y14	
Drug rehabilitation	Z503	
Drug abuse counselling and surveillance	Z715	
Drug use	Z722	
Personal history of psychoactive substance abuse	Z864	
Mental and behavioural disorders due to use of volatile solvents	F18	

Accidental poisoning by and exposure to noxious substances	X40–X44, X46-X49
Poisoning by chemical or noxious substance, undetermined intent	Y16-Y19
Special epileptic syndromes - (related to alcohol, drugs, etc)	G405
Blood-alcohol and blood-drug test	Z040
Alcohol-induced pseudo-Cushing's syndrome	244
Mental and behavioural disorders due to use of alcohol	F10
Degeneration of nervous system due to alcohol	G312
Alcoholic polyneuropathy	G621
Alcoholic myopathy	G721
Alcoholic cardiomyopathy	I426
Alcoholic gastritis	K292
Alcoholic liver disease	K70
Alcohol-induced acute pancreatitis	K852
Alcohol-induced chronic pancreatitis	K860
Finding of alcohol in blood	R780
Poisoning: antidotes and chelating agents, not elsewhere classified	T506
Toxic effect of alcohol	T51
Accidental poisoning by exposure to alcohol	X45
Poisoning by exposure to alcohol, undetermined intent	Y15
Evidence of alcohol involvement determined by blood alcohol level	Y90
Evidence of alcohol involvement determined by level of intoxication	Y91
Alcohol rehabilitation	Z502
Alcohol abuse counselling and surveillance	Z714
Alcohol use	Z721
**Mental Health conditions/ behavioural disorders**[2]	Coded from 5 years prior to index pregnancy to the start of pregnancy
Organic, including symptomatic, mental disorders	F00-F09
Schizophrenia, schizotypal and delusional disorders	F20-F29

Mood [affective] disorders	F30-F39	
Neurotic, stress-related and somatoform disorders	F40-F48	
Behavioural syndromes associated with physiological disturbances and physical factors	F50-F59	
Disorders of adult personality and behaviour	F60-F69	
Mental retardation	F70-F79	
Disorders of psychological development	F80-F89	
Behavioural and emotional disorders with onset usually occurring in childhood and adolescence	F90-F98	
Unspecified mental disorder	F99	
Sedatives, hypnotics and antianxiety drugs	Y47	
Psychotropic drugs, not elsewhere classified	Y49	
Personal history of other mental and behavioural disorders	Z865	
**Codes for previous pregnancy[3,4]**	ICD 10 codes: O342, O757, Z354, Z876 and Z875	Coded for from 2003 up to and include delivery episode
	OPCS-4 codes: R17–R25	Coded for from 2003 to start of pregnancy

*List of relevant medical conditions derived by RD and refined to be relevant to research question by DGB

ICD 10 Codes from studies:

1. Lee SI, Azcoaga-Lorenzo A, Agrawal U, Kennedy JI, Fagbamigbe AF, Hope H, Subramanian A, Anand A, Taylor B, Nelson-Piercy C, Damase-Michel C. Epidemiology of pre-existing multimorbidity in pregnant women in the UK in 2018: a population-based cross-sectional study. BMC pregnancy and childbirth. 2022 Feb 11;22(1):120.
2. Harron K, Gilbert R, Fagg J, Guttmann A, van der Meulen J. Associations between pre-pregnancy psychosocial risk factors and infant outcomes: a population-based cohort study in England. Lancet Public Health. 2021 Feb;6(2):e97-e105. doi: 10.1016/S2468-2667(20)30210-3. PMID: 33516292; PMCID: PMC7848754
3. Cromwell DA, Knight HE, Gurol-Urganci I. Parity derived for pregnant women using historical administrative hospital data: accuracy varied among patient groups. Journal of clinical epidemiology. 2014 May 1;67(5):578-85.
4. Sandall J, Murrells T, Dodwell M, Gibson R, Bewley S, Coxon K, et al. The efficient use of the maternity workforce and the implications for safety and quality in maternity care: a population-based, cross-sectional study. Health Serv Deliv Res 2014;2(38)

Table S3: Characteristics of women having severe maternal morbidity (SMM) stratified by IMD Quintile and (least to most deprived). N and %SMM in each category.

	**IMD Quintile**											
	**Most deprived 20%**		**More deprived 20-40%**		**Less deprived 40-60%**		**Less deprived 60-80%**		**Least deprived 80-100%**		**Total**	
	SMM		SMM		SMM		SMM		SMM		SMM	
	No	Yes	No	Yes	No	Yes	No	Yes	No	Yes	No	Yes
**Age group**												
<20												
N	30,408	378	16,535	227	10,652	147	6,934	104	4,113	53	68,642	909
%	98.77	1.23	98.65	1.35	98.64	1.36	98.52	1.48	98.73	1.27	98.69	1.31
20-25												
N	76,563	1,286	55,499	875	40,218	612	29,465	455	19,374	272	221,119	3,500
%	98.35	1.65	98.45	1.55	98.50	1.50	98.48	1.52	98.62	1.38	98.44	1.56
25-30												
N	80,364	1,497	80,931	1,371	73,334	1,155	66,410	1,000	53,394	780	354,433	5,803
%	98.17	1.83	98.33	1.67	98.45	1.55	98.52	1.48	98.56	1.44	98.39	1.61
30-35												
N	53,556	1,042	72,092	1,281	75,345	1,284	76,304	1,205	71,420	1,035	348,717	5,847
%	98.09	1.91	98.25	1.75	98.32	1.68	98.45	1.55	98.57	1.43	98.35	1.65
35-40												
N	20,485	426	29,125	561	30,695	552	30,188	523	28,655	470	139,148	2,532
%	97.96	2.04	98.11	1.89	98.23	1.77	98.30	1.70	98.39	1.61	98.21	1.79
>40												
N	4,373	119	5,673	135	5,856	128	5,988	126	5,589	119	27,479	627
%	97.35	2.65	97.68	2.32	97.86	2.14	97.94	2.06	97.92	2.08	97.77	2.23
**Ethnicity**												
White												
N	170,406	2,711	179,270	2,773	178,233	2,693	170,731	2,617	148,692	2,137	847,332	12,931
%	98.43	1.57	98.48	1.52	98.51	1.49	98.49	1.51	98.58	1.42	98.50	1.50
Black or Black British - Caribbean												
N	3,245	93	2,325	57	1,221	31	712	15	360	12	7,863	208
%	97.21	2.79	97.61	2.39	97.52	2.48	97.94	2.06	96.77	3.23	97.42	2.58
Black or Black British - African												
N	10,527	287	6,639	195	3,112	114	1,897	50	1,145	25	23,320	671
%	97.35	2.65	97.15	2.85	96.47	3.53	97.43	2.57	97.86	2.14	97.20	2.80
Asian or Asian British - Indian												
N	7,075	146	9,646	180	7,699	139	5,748	116	4,736	72	34,904	653
%	97.98	2.02	98.17	1.83	98.23	1.77	98.02	1.98	98.50	1.50	98.16	1.84
Asian or Asian British - Pakistani												
N	16,072	369	8,179	180	4,090	96	2,440	45	1,520	36	32,301	726

12

	**IMD Quintile**											
	**Most deprived 20%**		**More deprived 20-40%**		**Less deprived 40-60%**		**Less deprived 60-80%**		**Least deprived 80-100%**		**Total**	
	SMM		SMM		SMM		SMM		SMM		SMM	
	No	Yes	No	Yes	No	Yes	No	Yes	No	Yes	No	Yes
%	97.76	2.24	97.85	2.15	97.71	2.29	98.19	1.81	97.69	2.31	97.80	2.20
Asian or Asian British - Bangladeshi												
N	5,347	132	2,850	65	1,172	30	661	21	421	13	10,451	261
%	97.59	2.41	97.77	2.23	97.50	2.50	96.92	3.08	97.00	3.00	97.56	2.44
Mixed												
N	6,260	128	5,300	100	3,965	67	3,219	61	2,374	37	21,118	393
%	98.00	2.00	98.15	1.85	98.34	1.66	98.14	1.86	98.47	1.53	98.17	1.83
Other												
N	25,672	543	23,993	525	17,362	379	12,544	234	9,289	181	88,860	1,862
%	97.93	2.07	97.86	2.14	97.86	2.14	98.17	1.83	98.09	1.91	97.95	2.05
Missing												
	25,672	543	23,993	525	17,362	379	12,544	234	9,289	181	93,389	1,513
	97.93	2.07	97.86	2.14	97.86	2.14	98.17	1.83	98.09	1.91	98.41	1.59
**Pre-existing medical conditions (yes)**												
N	27,760	689	26,237	574	25,020	540	23,352	500	19,968	390	122,337	2,693
%	97.58	2.42	97.86	2.14	97.89	2.11	97.90	2.10	98.08	1.92	97.85	2.15
**Pre-existing mental health conditions (yes)**												
N	14,369	308	11,565	240	9,707	202	8,309	172	6,607	103	50,557	1,025
%	97.90	2.10	97.97	2.03	97.96	2.04	97.97	2.03	98.46	1.54	98.01	1.99
**Obesity/ overweight (yes)**												
N	63,417	1,437	53,752	1,173	45,616	989	39,643	825	30,454	629	232,882	5,053
%	97.78	2.22	97.86	2.14	97.88	2.12	97.96	2.04	97.98	2.02	97.88	2.12
**Substance misuse/smoking (yes)**												
N	32,885	595	26,572	464	22,230	383	18,989	356	14,162	209	114,838	2,007
%	98.22	1.78	98.28	1.72	98.31	1.69	98.16	1.84	98.55	1.45	98.28	1.72
**Total**												
N	265,749	4,748	259,855	4,450	236,100	3,878	215,289	3,413	182,545	2,729	1,159,538	19,218
%	98.24	1.76	98.32	1.68	98.38	1.62	98.44	1.56	98.53	1.47	98.37	1.63

*See list of conditions included list in Table S2

Table S4: Characteristics of women having severe maternal morbidity (SMM) stratified by ethnicity. N and %SMM in each category.

	**Ethnicity**																	
	**White**		**Black or Black British - Caribbean**		**Black or Black British - African**		**Asian or Asian British - Indian**		**Asian or Asian British - Pakistani**		**Asian or Asian British - Bangladeshi**		**Mixed**		**Other**		**Total**	
	SMM		SMM		SMM		SMM		SMM		SMM		SMM		SMM			
	No	Yes	No	Yes	No	Yes	No	Yes	No	Yes	No	Yes	No	Yes	No	Yes	No	Yes
**Age group**																		
<20																		
N	58,015	740	674	14	724	12	211	2	740	11	220	2	1,775	24	2,994	60	65,353	865
%	98.74	1.26	97.97	2.03	98.37	1.63	99.06	0.94	98.54	1.46	99.10	0.90	98.67	1.33	98.04	1.96	98.69	1.31
20-25																		
N	167,264	2,445	2,073	56	4,360	122	3,235	69	8,438	177	2,831	64	4,467	86	14,095	267	206,763	3,286
%	98.56	1.44	97.37	2.63	97.28	2.72	97.91	2.09	97.95	2.05	97.79	2.21	98.11	1.89	98.14	1.86	98.44	1.56
25-30																		
N	252,560	3,801	2,238	59	8,443	221	11,889	211	13,694	298	4,621	115	5,770	111	26,841	523	326,056	5,339
%	98.52	1.48	97.43	2.57	97.45	2.55	98.26	1.74	97.87	2.13	97.57	2.43	98.11	1.89	98.09	1.91	98.39	1.61
30-35																		
N	251,802	3,867	1,701	48	6,401	179	14,151	254	6,805	182	2,121	56	5,898	120	28,571	627	317,450	5,333
%	98.49	1.51	97.26	2.74	97.28	2.72	98.24	1.76	97.40	2.60	97.43	2.57	98.01	1.99	97.85	2.15	98.35	1.65
35-40																		
N	98,697	1,671	903	29	2,697	100	4,594	91	2,150	49	546	15	2,685	43	13,481	297	125,753	2,295
%	98.34	1.66	96.89	3.11	96.42	3.58	98.06	1.94	97.77	2.23	97.33	2.67	98.42	1.58	97.84	2.16	98.21	1.79
>40																		
N	18,994	407	274	2	695	37	824	26	474	9	112	9	523	9	2,878	88	24,774	587
%	97.90	2.10	99.28	0.72	94.95	5.05	96.94	3.06	98.14	1.86	92.56	7.44	98.31	1.69	97.03	2.97	97.69	2.31
**IMD Quintile**																		
Most deprived 20%																		
N	170,406	2,711	3,245	93	10,527	287	7,075	146	16,072	369	5,347	132	6,260	128	25,672	543	244,604	4,409
%	98.43	1.57	97.21	2.79	97.35	2.65	97.98	2.02	97.76	2.24	97.59	2.41	98.00	2.00	97.93	2.07	98.23	1.77
More deprived 20-40%																		
N	179,270	2,773	2,325	57	6,639	195	9,646	180	8,179	180	2,850	65	5,300	100	23,993	525	238,202	4,075
%	98.48	1.52	97.61	2.39	97.15	2.85	98.17	1.83	97.85	2.15	97.77	2.23	98.15	1.85	97.86	2.14	98.32	1.68
Less deprived 40-60%																		
N	178,233	2,693	1,221	31	3,112	114	7,699	139	4,090	96	1,172	30	3,965	67	17,362	379	216,854	3,549
%	98.51	1.49	97.52	2.48	96.47	3.53	98.23	1.77	97.71	2.29	97.50	2.50	98.34	1.66	97.86	2.14	98.39	1.61
Less deprived 60-80%																		
N	170,731	2,617	712	15	1,897	50	5,748	116	2,440	45	661	21	3,219	61	12,544	234	197,952	3,159
%	98.49	1.51	97.94	2.06	97.43	2.57	98.02	1.98	98.19	1.81	96.92	3.08	98.14	1.86	98.17	1.83	98.43	1.57
Least deprived 80-100%																		

	Ethnicity																	
	**White**		**Black or Black British - Caribbean**		**Black or Black British - African**		**Asian or Asian British - Indian**		**Asian or Asian British - Pakistani**		**Asian or Asian British - Bangladeshi**		**Mixed**		**Other**		**Total**	
	SMM		SMM		SMM		SMM		SMM		SMM		SMM		SMM			
N	148,692	2,137	360	12	1,145	25	4,736	72	1,520	36	421	13	2,374	37	9,289	181	168,537	2,513
%	98.58	1.42	96.77	3.23	97.86	2.14	98.50	1.50	97.69	2.31	97.00	3.00	98.47	1.53	98.09	1.91	98.53	1.47
**Pre-existing Medical Conditions***																		
N	104,423	2,102	1,113	42	1,530	86	2,371	66	2,561	103	721	26	2,262	50	5,349	176	120,330	2,651
%	98.03	1.97	96.36	3.64	94.68	5.32	97.29	2.71	96.13	3.87	96.52	3.48	97.84	2.16	96.81	3.19	97.84	2.16
**Pre-existing Mental Health Conditions***																		
N	45,272	861	368	18	395	18	447	16	715	22	229	6	876	22	1,602	47	49,904	1,010
%	98.13	1.87	95.34	4.66	95.64	4.36	96.54	3.46	97.01	2.99	97.45	2.55	97.55	2.45	97.15	2.85	98.02	1.98
**Substance misuse or smoking8**																		
N	102,685	1,710	768	24	682	27	1,018	26	1,411	39	419	11	2,272	53	3,715	92	112,970	1,982
%	98.36	1.64	96.97	3.03	96.19	3.81	97.51	2.49	97.31	2.69	97.44	2.56	97.72	2.28	97.58	2.42	98.28	1.72
**Obesity or overweight***																		
N	181,864	3,588	2,078	85	6,002	211	4,665	111	6,720	191	1,830	72	4,215	115	12,533	376	219,907	4,749
%	98.07	1.93	96.07	3.93	96.60	3.40	97.68	2.32	97.24	2.76	96.21	3.79	97.34	2.66	97.09	2.91	97.89	2.11
**Total**																		
N	847,332	12,931	7,863	208	23,320	671	34,904	653	32,301	726	10,451	261	21,118	393	88,860	1,862	1,066,149	17,705
%	98.50	1.50	97.42	2.58	97.20	2.80	98.16	1.84	97.80	2.20	97.56	2.44	98.17	1.83	97.95	2.05	98.37	1.63

*See list of conditions included list in Table S2

Table S5. Number and the percentage N(%) of total severe maternal morbidity breakdown by diagnosis/ procedure of the EMMOI

**Diagnosis**	**N(%)**
Acute abdomen	68 (0.01)
Acute renal failure	5,871 (0.50)
Acute psychosis	149 (0.01)
Cardiac arrest/ failure or infarction	678 (0.06)
Cerebral oedema/coma	43 (0.00)
Disseminated Intravascular Coagulation	21 (0.00)
Cerebrovascular accident	58 (0.00)
Major complications of anaesthesia	0 (0.00)
Obstetric Embolism (including Amniotic embolism)	515 (0.04)
Shock	507 (0.04)
Sickle cell anaemia with crisis	54 (0.00)
Status asthmaticus	64 (0.01)
Status epilepticus	22 (0.00)
Uterine rupture	443 (0.04)
Eclampsia	873 (0.07)
Sepsis	8,691 (0.74)
Cerebral Venous Thrombosis	2 (0.00)
**Procedures**	
Assisted ventilation including tracheostomy	404 (0.03)
Curettage in combination with a general anaesthetic	0 (0.00)
Dialysis	55 (0.00)
Evacuation of haematoma	1,096 (0.09)
Hysterectomy	68 (0.01)
Procedures to reduce blood flow to uterus	85 (0.01)
Re-closure of disrupted caesarean section wound	292 (0.02)
Repair of bladder or cystostomy	229 (0.02)
Repair of intestine	53 (0.00)

Table S6: Average adjusted predictions of SMM for IMD Quintile and Ethnicity based on the final multivariable model (Model 3) adjusted for age, substance misuse, pre-existing medical conditions, pre-existing mental health conditions and obesity

	Average adjusted predictions [Proportion of women with SMM with 95% Confidence Intervals]
**IMD Quintile**	
Most deprived 20%	0.017
	[0.016 0.017]
More deprived 20-40%	0.017
	[0.016 0.018]
Less deprived 40-60%	0.016
	[0.016 0.017]
Less deprived 60-80%	0.016
	[0.015 0.017]
Least deprived 80-100%	0.015
	[0.015 0.016]
**Ethnicity**	
White	0.015
	[0.015 0.015]
Black or Black British - Caribbean	0.025
	[0.022 0.028]
Black or Black British - African	0.028
	[0.026 0.03]
Asian or Asian British - Indian	0.019
	[0.018 0.021]
Asian or Asian British - Pakistani	0.022
	[0.021 0.024]
Asian or Asian British - Bangladeshi	0.025
	[0.022 0.028]
Mixed	0.018
	[0.016 0.020]
Other	0.021
	[0.020 0.022]

Geddes-Barton D, *et al. J Epidemiol Community Health* 2024; 78:500–507. doi: 10.1136/jech-2024-222060

Table S7. Sensitivity analysis after excluding women with parity coded as missing in the 'maternity tail' – 510,439 women (43.30%) and after multiple imputation if data missing on ethnicity (FMI 11.75%)

	**Multiple Imputation – fully adjusted model (Model 3)**	**Excluding women with parity coded as missing in the maternity tail – fully adjusted model (Model 3)**
	Odds ratios [95% Confidence Intervals]	Odds ratios [95% Confidence Intervals]
**IMD Quintile**		
Most deprived 20%	1.12	1.13
	[1.06 1.17]	[1.05 1.21]
More deprived 20-40%	1.10	1.06
	[1.04 1.15]	[0.99 1.14]
Less deprived 40-60%	1.08	1.05
	[1.03 1.13]	[0.98 1.12]
Less deprived 60-80%	1.05	1.04
	[1.00 1.11]	[0.97 1.12]
Least deprived 80-100%	1[ref]	1[ref]
**Age group**		
<20	0.86	0.92
	[0.80 0.92]	[0.84 1.02]
20-35	1[ref]	1[ref]
25-30	1.05	1.02
	[1.01 1.10]	[0.96 1.08]
30-35	1.12	1.07
	[1.07 1.16]	[1.00 1.13]
35-40	1.19	1.18
	[1.13 1.26]	[1.10 1.27]
>40	1.45	1.52
	[1.33 1.58]	[1.35 1.73]
**Ethnicity**		
White	1[ref]	1[ref]
Black or Black British - Caribbean	1.68	1.79
	[1.47 1.94]	[1.49 2.14]
Black or Black British - African	1.87	1.80
	[1.73 2.03]	[1.62 2.01]
Asian or Asian British - Indian	1.28	1.29
	[1.18 1.39]	[1.17 1.43]
Asian or Asian British - Pakistani	1.51	1.47
	[1.40 1.63]	[1.32 1.64]
Asian or Asian British - Bangladeshi	1.70	1.72
	[1.50 1.93]	[1.49 2.00]
Mixed	1.21	1.12
	[1.10 1.35]	[0.97 1.29]
Other	1.41	1.41
	[1.34 1.48]	[1.32 1.50]
**Pre-existing Medical Conditions***	1.36	1.33
	[1.30 1.42]	[1.25 1.41]
**Pre-existing Mental Health Condition***	1.16	1.10
	[1.08 1.24]	[1.00 1.21]
**Substance Misuse or Smoking***	0.98	1.01
	[0.94 1.02]	[0.94 1.08]
**Obesity/ Overweight***	1.41	1.40
	[1.37 1.46]	[1.33 1.46]

*See list of conditions included list in Table S2

Geddes-Barton D, *et al. J Epidemiol Community Health* 2024; 78:500–507. doi: 10.1136/jech-2024-222060

Table S8: E-values

	E value (point estimate)	E value (Confidence Interval)
**IMD Quintile**		
Most deprived 20%	1.513	1.344
More deprived 20-40%	1.432	1.244
Less deprived 40-60%	1.364	1.163
Less deprived 60-80%	1.312	1.110
Least deprived 80-100%	1(ref)	1(ref)
**Ethnicity**		
White	1(ref)	1(ref)
Black or Black British - Caribbean	2.707	2.236
Black or Black British - African	3.083	2.791
Asian or Asian British - Indian	1.832	1.591
Asian or Asian British - Pakistani	2.344	2.104
Asian or Asian British - Bangladeshi	2.749	2.323
Mixed	1.762	1.459
Other	2.170	2.015

References

1. Haines N. Births in England and Wales: 2017 [Internet]. Births in England and Wales - Office for National Statistics. Office for National Statistics; 2018 (accessed June 2, 2022). Available from: https://www.ons.gov.uk/peoplepopulationandcommunity/birthsdeathsandmarriages/livebirths/bulletins/birthsummarytablesenglandandwales/2017
2. Cromwell DA, Knight HE, Gurol-Urganci I. Parity derived for pregnant women using historical administrative hospital data: accuracy varied among patient groups. Journal of clinical epidemiology. 2014 May 1;67(5):578-85.
3. McLennan D, Noble S, Noble M, Plunkett E, Wright G, Gutacker N. The English indices of deprivation 2019: technical report.
4. ETHNIC CATEGORY [Internet]. www.datadictionary.nhs.uk (accessed June 2, 2022). Available from: https://www.datadictionary.nhs.uk/data_elements/ethnic_category.html
5. Draper, E., Manktelow, B., Smith, P., Kurinczuk, J., Matthews, R., Smith, L., Gallimore, I. (2022). MBRRACE-UK Perinatal Mortality Surveillance Report: UK Perinatal Deaths for Births from January to December 2020. Oxford: National Perinatal Epidemiology Unit, University of Oxford.
6. Nair M, Kurinczuk JJ, Knight M. Establishing a national maternal morbidity outcome indicator in England: a population-based study using routine hospital data. PLoS One. 2016 Apr 7;11(4):e0153370
7. Roberts CL, Cameron CA, Bell JC, Algert CS, Morris JM. Measuring maternal morbidity in routinely collected health data: development and validation of a maternal morbidity outcome indicator. Medical care. 2008 Aug 1:786-94

8. Snelgrove JW, Lam M, Watson T, Richard L, Fell DB, Murphy KE, Rosella LC. Neighbourhood material deprivation and severe maternal morbidity: a population-based cohort study in Ontario, Canada. BMJ open. 2021 Oct 1;11(10):e046174
9. Schoenaker DAJM, Stephenson J, Smith H, Thurland K, Duncan H, Godfrey KM, et al. Women's preconception health in England: a report card based on cross-sectional analysis of national maternity services data from 2018/2019. *BJOG*. 2023; 130(10): 1187–1195. https://doi.org/10.1111/1471-0528.17436
10. Boyd A, Cornish R, Johnson L, Simmonds S, Syddall H, Westbury L. Understanding Hospital episode statistics (HES). London, UK: CLOSER. 2017
